# Cellular and environmental dynamics influence species-specific extents of organelle gene retention

**DOI:** 10.1098/rspb.2022.2140

**Published:** 2023-03-08

**Authors:** Belén García Pascual, Jan M. Nordbotten, Iain G. Johnston

**Affiliations:** ^1^ Department of Mathematics, University of Bergen, Bergen, Norway; ^2^ Computational Biology Unit, University of Bergen, Bergen, Norway

**Keywords:** organelle DNA, evolution, environmental dynamics, mitochondria, chloroplasts‌, organelle evolution

## Abstract

Mitochondria and plastids rely on many nuclear-encoded genes, but retain small subsets of the genes they need to function in their own organelle DNA (oDNA). Different species retain different numbers of oDNA genes, and the reasons for these differences are not completely understood. Here, we use a mathematical model to explore the hypothesis that the energetic demands imposed by an organism’s changing environment influence how many oDNA genes it retains. The model couples the physical biology of cell processes of gene expression and transport to a supply-and-demand model for the environmental dynamics to which an organism is exposed. The trade-off between fulfilling metabolic and bioenergetic environmental demands, and retaining genetic integrity, is quantified for a generic gene encoded either in oDNA or in nuclear DNA. Species in environments with high-amplitude, intermediate-frequency oscillations are predicted to retain the most organelle genes, whereas those in less dynamic or noisy environments the fewest. We discuss support for, and insight from, these predictions with oDNA data across eukaryotic taxa, including high oDNA gene counts in sessile organisms exposed to day-night and intertidal oscillations (including plants and algae) and low counts in parasites and fungi.

## 1. Introduction

Most eukaryotic cells contain bioenergetic organelles: mitochondria and, in the case of photosynthetic organisms, plastids (including the particular case of chloroplasts). These organelles were originally independent bacteria with their own genomes, and through the evolutionary history of endosymbiosis, they have transferred most of their genes to the nuclear genome of the host cell or lost them completely [1–6]. The transfer of genetic material for organelle to nucleus is ongoing in several species [7,8]; experiments have characterized the rate of this process in plants [9] and several molecular mechanisms have been proposed for the transfer process [7]. However, a common (but not universal) feature of present-day eukaryotes is that they have retained a small subset of their genes in their own organelle DNA (oDNA), with oDNA in different species containing strikingly different gene counts [1,10–12].

Transfer to the nucleus provides some genetic advantages to organelle genes that are absent in the organelle genome [1,5]. Nuclear encoding helps avoid sources of mutation in the organelle compartments, which include chemical damage from free radicals [13] and replicative errors [14] (which may be more dominant [15]). Nuclear encoding also helps the avoidance of Muller’s ratchet (the irreversible process of accumulation of deleterious mutations) present in the organelles [10,16] and allows sexual recombination and DNA repair in the nucleus [13]. Given these advantages, the question of why bioenergetic organelles retain the genes that they do has been debated for years. At the most fundamental level, there are two pertinent subquestions. The first, gene-centric, question is what makes a given gene more or less likely to be retained in oDNA. The second, species-centric, question is what makes a given species more or less likely to retain a higher or lower number of oDNA genes.

The first question, why a given gene is more or less likely to be retained in oDNA, has had several potential answers proposed over time (summarized in Giannakis *et al.* [11]). It has recently been shown that a combination of these can help explain gene-specific patterns of oDNA retention across organelles and eukaryotes [11]. Of particular note here are the hydrophobicity hypothesis [17] and the co-localization for redox regulation (CoRR) hypothesis [13,18–21]. The hydrophobicity hypothesis asserts that hydrophobic gene products are harder to import to the organelle from the outside (either due to translocation into the organelle [1,22] or mistargeting [17,23], proposing that organelle genes encoding hydrophobic gene products are thus more likely to be retained in the organelles. The CoRR hypothesis proposes that genes are retained in oDNA to allow local, tight control of the energetic machinery [20,21], so that organelles can better adapt to imposed energetic demands. This idea is supported by the importance of retained oDNA genes in controlling redox processes [2,11,21].

The second question, why some species retain more organelle genes than others, remains more open. There exists substantial diversity in oDNA gene counts across eukaryotes [3,11]. Some jakobid protists retain over 60 protein-coding mitochondrial DNA (mtDNA) genes, plants retain fewer and metazoa fewer still with a common 13-protein gene profile shared by a large majority of taxa (including humans). The highest plastid gene counts found so far appear in the group of red alga Rhodophyta with over 200 protein-coding plastid DNA (ptDNA) genes and up to 35 protein-encoding mtDNA genes. By contrast, parasitic species (notably including alveolates) contain very few protein-coding mtDNA and ptDNA genes and some have even lost mtDNA entirely [24].

Some specific instances of this diversity have theoretical explanations. Parasitic organisms, for example, can hijack metabolic and energetic budgets of their hosts, so presumably, organelle genes are lost since their bioenergetic organelles have fewer required functions. Self-pollinating and clonal plant species have transferred more mtDNA genes to the nucleus than outcrossing plants [25], which has been theoretically explained by the acceleration of beneficial transfer by self-pollination [26]. But how can the diversity of oDNA gene counts in other taxa be explained?

To address this species-specific question, we focus on how the energetic demand imposed by the environment changes over time. Following the CoRR idea that retaining genes in oDNA improves organelles’ local responses to changing conditions, we hypothesize that organisms facing large and/or rapid environmental changes in bioenergetic demand require more local control over organelle machinery to respond to these changes [27]. The hypothesis suggests that organisms in stable and low-demand environments (including parasites) require less organelle control, as the production of energy is not so challenging. Organisms subject to more dynamic environmental demands (for example, diurnal oscillations of light or semidiurnal oscillations of tide) require tighter control over challenging energetic and metabolic demands, so these organisms are predicted to retain a higher count of oDNA genes.

Hypotheses about evolutionary processes can be challenging to test with experiments. Quantitative modelling can shed light on evolutionary pressures and dynamics, as demonstrated by powerful theoretical studies exploring the coevolution of oDNA and the host cell [28–32], and models of the specific features involved in oDNA gene retention [11,33], although few quantitative models to our knowledge have explored CoRR in the same depth. Here, to test our hypothesis, we propose and analyse a mathematical model for a given organelle gene in a given organism. This model captures the interplay of cellular processes (like gene expression, import of gene product to the organelle and degradation), environmental dynamics (like the amplitude and frequency of an environmental wave, also understood as the energetic demand placed on the organism) and the proportion of wild-type oDNA which allows the organelle to functionally synthesize the gene products it needs. We use the model to probe how environmental dynamics influence the organism’s ability to meet energetic demands for each of the two cases of encoding compartments: the nucleus and the organelle, providing a general quantitative framework to explore links between CoRR, environmental dynamics and gene retention.

## 2. Methods

### (a) Model description

**Figure 1.**
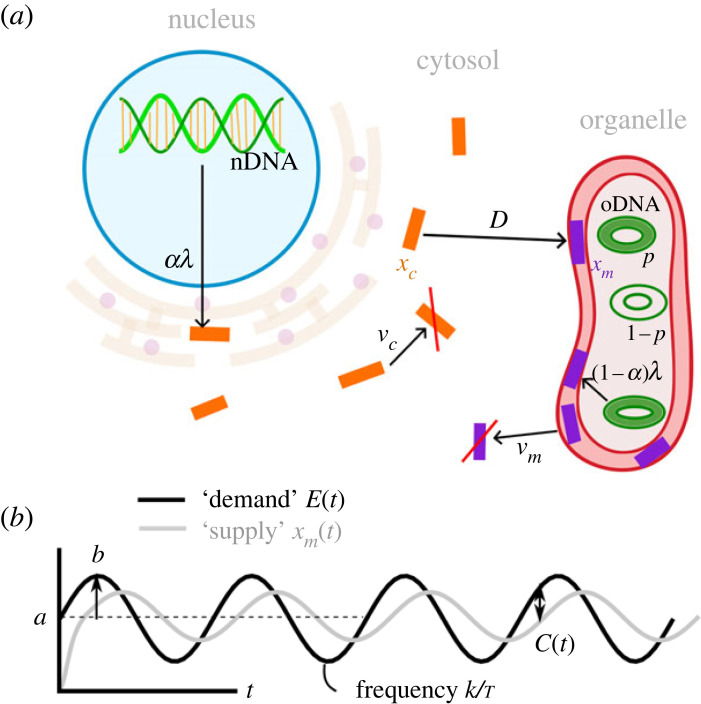
Model outline. (*a*) The cell biological processes modelled in gene expression and translocation. A gene encoded in the organelle (corresponding to model index *α* = 0) is expressed with rate *λ* from wild-type oDNA. The proportion of functional, wild-type oDNA is *p*, with the remaining proportion assumed to be mutationally damaged and incapable of producing functional machinery. An organelle gene encoded in the nucleus (corresponding to model index *α* = 1) is expressed from nDNA with rate *λ*, and its gene product is imported to the organelle with a transport rate *D*. The labels *x*_*c*_ and *x*_*m*_ represent the amount of gene product in the cytosol and organelle, respectively, and *ν*_*c*_ and *ν*_*m*_ are degradation rates in those compartments. (*b*) Environmental supply and demand. The environment places demand on the organelle machinery. For oscillating signals, this demand has mean *a*, relative amplitude *b* and frequency *k*/*τ*. Organelle gene products *x*_*m*_ will respond to this demand: following some transient behaviour an oscillation will also occur. An instantaneous cost *C*(*t*) is incurred, being the absolute difference between environmental demand *E*(*t*) and functional machinery supply *x*_*m*_(*t*).

The mathematical model explores the ability of an organism to meet environmental demands, considering the interplay of cell biological processes and particular model environments. It is summarized in figure 1 and built up in stages as follows.

#### (i) Cell biological processes: gene expression, transport and degradation

The setup of the model is for a generic gene encoding organelle machinery. The gene may be encoded in the organelle, or in the nucleus of the cell, in which case it must be transported to the organelle through the cytosol. We assume that there is no systematic difference in the intrinsic properties of this representative gene between the two possible encoding compartments, neglecting, for example, differences in genetic code that may be required for the different locations [7]. Working at a coarse-grained level, we will use this general picture to describe both mitochondria and plastids. The model has two dynamic variables: *x*_*m*_(*t*) is the available amount of functional gene product in the organelle at time *t* (for example, the various protein subunits of electron transport chain complexes) and *x*_*c*_(*t*) is the available amount of gene product in the cytosol at time *t*.

The parameters of the model for the cellular processes include the following non-negative constant rates: *λ* is the baseline synthesis rate of gene product (which will be scaled by the cell’s response to the environment, see below), *D* is the import or transport rate of gene product from the cytosol to the organelle, *ν*_*m*_, *ν*_*c*_ are the degradation rates of gene product in the organelle and in the cytosol, respectively. We also include a parameter *p*, the proportion of wild-type oDNA which allows the synthesis of functional gene product in the organelle. This parameter is required to capture the potential for oDNA damage, which occurs on longer (evolutionary) time scales than the cell biological processes above. Instead of modelling both gene expression and mutation time scales explicitly, which would require a rather more involved simulation setup, we coarse-grain the effect of mutation into this expected oDNA damage load which stays constant over a cell lifetime. This damage can be pictured as arising from mutation in the history of the lineage of the cell; the purpose of our study here is to characterize the balance between this potential accumulation of oDNA damage and the selective pressure favouring oDNA encoding. Lower *p* corresponds to more oDNA damage, compromising the expression of functional organelle machinery; *p* = 1 corresponds to perfect oDNA and hence the maximum possible expression capacity. The propensity for oDNA damage in an organism, both within a lifetime and across generations, will act to reduce *p*. The cell processes and properties described by these parameters are illustrated in figure 1*a*.

The parameters *D* and *ν*_*c*_ describe the ability of a nuclear-encoded gene product to translocate to its required position in the organelle. These can be used to model different mechanisms proposed for the hydrophobicity hypothesis. One mechanism is that hydrophobic gene products cannot readily be unpacked to import into the organelle [1,22], corresponding to a high value of the degradation rate in the cytosol *ν*_*c*_, as the gene product is merely lost and hence can be considered as degraded. Another mechanism is that the gene product is mistargeted, usually to the endoplasmic reticulum [17,23], so that the import to the organelle takes a much longer time: corresponding to a low value of the transport rate *D*.

#### (ii) Coupling of the environmental supply-and-demand model and the cell biology processes

We denote by *E*(*t*) the energetic demand placed on the cell by the environment. The goal of the cell is to match *x*_*m*_(*t*), the ‘supply’ of gene product present in the organelle, to environmental demand *E*(*t*). We picture the cell as sensing, and able to respond to, environmental demands by expressing genes that support organelle function. Hence, we define a feedback signalling function *f*(*E*(*t*), *x*_*m*_(*t*)) that controls the production of gene product as

2.1 $$f\;(E(t),x_{m}(t))=\left\{ \begin{array}{cc} E(t)-x_{m}(t)\; & \mathrm{if}\;x_{m}(t)<E(t)\;\\ 0\; & \mathrm{if}\;x_{m}(t)\geq E(t).\; \end{array}\right.$$

Since the dynamics depend on the compartment where the organelle gene is encoded (the organelle itself or the nucleus), we use a coefficient *α* as a model index representing the encoding compartment, where *α* = 0 if the gene is in the organelle or *α* = 1 if the gene is in the nucleus.

We then have our model of first-order, linear ordinary differential equations describing the instantaneous rates of change of the variables *x*_*m*_(*t*) and *x*_*c*_(*t*) as

2.2 $$\begin{array}{cc} & \frac{dx_{m}}{dt}=(1-\alpha )\lambda pf(E,x_{m})+Dx_{c}-\nu_{m}x_{m}\\ \mathrm{and}\; & \frac{dx_{c}}{dt}=\alpha \lambda f(E,x_{m})-Dx_{c}-\nu_{c}x_{c} \end{array}\}$$

for either *α* = 0 or *α* = 1. The right-hand-side terms respectively describe gene expression in response to environmental demand, transport from the cytoplasm to the organelle, and degradation of gene products. We take initial conditions (*x*_*m*_(0), *x*_*c*_(0)) = (0, 0), but we focus on long-term behaviour after the corresponding transient periods have disappeared, described below.

The mathematical properties of equation (2.2) are studied in the electronic supplementary material, text A.1-A.3, including well-posedness (A.1) together with existence and stability of equilibrium points (A.2 and A.3).

#### (iii) Bioenergetic cost

The model is of supply-and-demand nature by equation (2.1), so as in previous quantitative work [34], we can define a cost function that measures how well the organelle supplies the organism with the energetic and/or metabolic requirements that its environment demands. A high cost corresponds to supply far away from the demand, either surplus or deficit. This cost function depends on the specific encoding compartment, so that the compartment incurring the lowest cost is interpreted as the most favourable in which to encode the gene. We define the cost function as the absolute difference between environmental demand *E*(*t*) and supply of functional gene product in the organelle *x*_*m*_(*t*) integrated over the time window [*t*_*i*_, *t*_*f*_]:

2.3 $$c_{\alpha }(t_{i},t_{\;f})=\int_{t_{i}}^{t_{\;f}}|E(t)-x_{m}(t)|\;dt.$$

This supply-and-demand model is illustrated in figure 1*b*. To ensure the robustness of our results, we consider different choices of cost functions (electronic supplementary material, figures S4, S13 and S14) and show that the model’s behaviour is similar across several choices.

#### (iv) Specific types of environmental demands

We explore the extent to which an organism is likely to retain oDNA genes by looking at the influences that different types of environmental demands *E*(*t*) have on the system. We consider static environments, periodically changing environments and randomly changing environments.

Static environments are simply modelled with *E*(*t*) = *a*. For the case of a periodically changing environment, we let *a* > 0 be the time-averaged value of environmental demand, *ab* the amplitude of the oscillation (with *b* seen as the relative amplitude), *τ* the characteristic time-scale of the system (for example, a day in a physical context) and *k* the frequency of the oscillation relative to the time scale *τ* (figure 1*b*). We then model the energetic demand placed on the organism by a periodically changing environment as a wave *E*(*t*) = *E*_*p*_(*t*) where *E*_*p*_(*t*) is defined as

2.4 $$E_{\;p}(t)=a\left(1+b\;\sin\left(\frac{2\pi kt}{\tau }\right)\right)\;\text{for}\;b\in [0,1].$$

For a randomly changing environment, we consider *E*(*t*) as a stochastic process which is either uncorrelated white noise (as used in descriptions of terrestrial environments [35]), or correlated red noise (as used in descriptions of marine and coastal environments [35]), also called a random walk.

For numerical implementation of the noisy environments, for a time interval [0,Γ] of the model, we define a partition $0=s_{0}<s_{1}<\ldots <s_{k-1}<s_{k}=\Gamma$ so that *D* = {*d*_*i*_}_*i*∈*I*_, where *d*_*i*_ = [*s*_*i*_, *s*_*i*+1_], is the collection of time subintervals. We then consider *D* to be the time-discrete, finite and bounded domain of the time-continuous series of white and red noise. The range of both types of noise is [0, 2*a*], so that the average value is *a* as for the periodically changing environment (equation (2.4)).

For each *i*, we draw a random number *u*_*i*_ that has a uniform distribution in [0, 2*a*], i.e. *u*_*i*_ ∼ *U*(0, 2*a*). White noise *E*_*w*_(*t*) is then defined as

2.5 $$E_{w}(t)=u_{i}\;\text{for}\;t\in d_{i}.$$

To define red noise *E*_*r*_(*t*), we write the accumulation of *u*_*i*_ with a step-size |*d*_*i*_| = *s*_*i*+1_ − *s*_*i*_, and we keep it bounded in [0, 2*a*] as

$$w_{i}=\max(0,\min(2a,w_{i-1}+|d_{i}|(u_{i}-a))),$$

then we define *E*_*r*_(*t*) as

2.6 $$E_{r}(t)=w_{i}\;\mathrm{for}\;t\in d_{i}.$$

### (b) Biological parameterizations

The specific values of the rates involved in our model vary substantially across species, and across genes in the same species. However, we can both connect with biological quantities at a coarse-grained level to interpret our model in an informative way [36], and also scan through plausible ranges of the parameters involved to explore the range of possible behaviours of system under different biological cases.

We take characteristic rates measured for the case of yeast *Saccharomyces cerevisiae* as reference for our model. The synthesis rate of gene product is a combination of transcription and translation, the rates of which vary substantially across genes; we will explore orders-of-magnitude ranges in these parameters throughout this study. As first estimates, a transcription rate of 0.12 min^−1^ messenger RNA molecules [37] and a translation rate of 0.43 min^−1^ protein molecules [38] give an overall rate of around 0.1 min^−1^. We will investigate our model’s behaviour over a range of several orders of magnitude here, but we begin by choosing a general time scale that is within a typical range for these processes, choosing *T* = 10 min to be the time unit in our model, so that the synthesis rate is set as *λ* = 1 *T*^−1^ for simulations by default (the effect of different synthesis rates are shown in electronic supplementary material, figures S11 and S12). The average scale of protein half-lives in the cytosol of 43 min [39] gives *ν*_*c*_ ≈ 0.1 *T*^−1^. Protein half-lives in the mitochondrion vary dramatically across hours and days for different genes, suggesting a possible range of *ν*_*m*_ ≈ 0.01 − 0.1 *T*^−1^ [40]. Import rates to the mitochondrion also vary substantially across genes, suggesting *D* ≈ 0.1–10 *T*^−1^ [41]. The model parameters that we map therefore contain a range of values that correspond to plausible dynamics for real genes. Different eukaryotic species will have different values for these parameters, but the ranges we consider are compatible with observations across kingdoms (for example, [42]).

We set a time window [0, 2 × 144] of 2 days in *T* time units for the numerical simulations. To remove the transient periods determined by the initial conditions (*x*_*m*_(0), *x*_*c*_(0)) = (0, 0) and focus on the equilibrium phases of the system, we use the first day [0, 144] as an equilibration step. Then we take the solutions of the model and compute the respective cost functions (equation (2.3)) only for the last full day, which corresponds to the time window [144, 2 × 144].

In the periodic environment *E*_*p*_(*t*) (equation (2.4)), we set the characteristic time-scale *τ* to be a full 24 h period in *T* units, i.e. *τ* = 144*T* (*τ* = 144*T* = 144 × 10 min = 24 h). So *k* = 1 corresponds to diurnal oscillation (as with day-night cycles), and *k* = 2 to semidiurnal oscillation (as with tides).

The wild-type proportion of oDNA *p* is a coarse-grained measure of oDNA integrity in our model as described above. Rather than attempting to set this value to match a given biological instance, we will explore the behaviour of the system as it varies. *p* can be pictured as a snapshot value from the ongoing process of oDNA damage accumulation, segregation and mitigation [43,44].

### (c) Numerical implementation

The numerical simulations and visualizations of results were all performed in Python. The model of ordinary differential equations presented in equation (2.2) was solved using the numerical integrator scipy.integrate.odeint that uses the method LSODA from the FORTRAN library odepack [45,46]. The heatmaps were made using seaborn [47], and the time-lines and phase portraits were made using matplotlib.pyplot [48]. The integral of the cost function (equation (2.3)) was computed using the composite Simpson’s rule using scipy.integrate.simps. Mathematical expressions were implemented using NumPy [49] and mpmath [50]. The code can be found in https://github.com/StochasticBiology/Environmental-oDNA-retention.git.

### (d) Bioinformatics

The curation of oDNA gene counts follows and builds upon the pipeline for eukaryotic oDNA analysis in [11]. All available complete mtDNA and ptDNA sequences were downloaded from RefSeq [51]. Gene annotations were systematized with BioPython [52] according to a manually-curated list of label substitutions, taken from and validated in [11]. The subset of protein-coding genes in these lists (omitting RNA genes, gene fragments, open reading frames not known to encode a protein and various other anomalous entries) present in each species’ oDNA was then recorded. The species in the dataset were embedded in a Common Taxonomy Tree [53] and the members of each basal eukaryotic clade compiled into a corresponding set using R [54], with libraries ggplot2 [55], gridExtra [56] and phytools [57].

## 3. Results

Our model, described in the Methods, pictures the environment as imposing metabolic and bioenergetic demands on the cell, which may be static or vary periodically or randomly over time. This may correspond, for example, to diurnal variation in light levels, temperature, animal activity and so on; to semidiurnal tidal variation in oxygenation and salinity; or to more rapid variation due to bursts of activity, fluctuating shade or other conditions. The cell expresses organelle genes to produce gene products in an attempt to supply the required machinery to meet this demand. Genes may be encoded in either the nucleus, in which case their gene products must be imported to the organelle, or in the organelle, in which case the genes are potentially subject to mutational damage. We integrate the absolute difference between supply and demand over a simulated time window to calculate the cost for each compartment (equation (2.3)) for a given parameterization.

There are several parameters in the model corresponding to cell biological and environmental quantities [58]. Some key parameters are the synthesis of gene product *λ*, the transport *D* and the degradation of gene product in the organelle *ν*_*m*_ and in the cytosol *ν*_*c*_. We also consider a measure of oDNA integrity, interpreted as the proportion *p* of wild-type, functional oDNA. Rather than attempt an exhaustive characterization of the full parameter space in the main text, we focus on several cases which illustrate the more general trends, and support these examples with other results in the electronic supplementary material, information. We explore biologically plausible ranges of parameters, with simulation time unit *T* = 10 min setting the scale for other parameters (see Methods), and simulating for a time scale of a full day after allowing transient behaviours to disappear. Across these ranges, we report how the balance between environmental response and mutational load changes with the relative values of the model parameters, rather than focussing on specific absolute values (which change within and between cells and species).

### (a) Static environments

To explore the influences of the different cell biological parameters on the model, we first look at the case of a static environment, where *E*(*t*) = *a* for all *t*. In electronic supplementary material, text A.2, we algebraically find the solutions of the system when it is in equilibrium, and from there we can directly compute the instantaneous cost $C_{\alpha }$ for encoding compartment *α* as the absolute difference between the energetic demand *E*(*t*) = *a* and the supply of gene product in the organelle *x*_*m*_(*t*). The ratio between the instantaneous cost for the organelle-encoding strategy (*α* = 0) and the nuclear-encoding strategy (*α* = 1) is

3.1 $$\begin{array}{r} \frac{C_{0}}{C_{1}}=\frac{a-(\lambda p/(\lambda p+\nu_{m}))a}{a-(\lambda D/(\lambda D+(D+\nu_{c})\nu_{m}))a}=\frac{\lambda D+(D+\nu_{c})\nu_{m}}{\left(\lambda p+\nu_{m})(D+\nu_{c}\right)}. \end{array}$$

**Figure 2.**
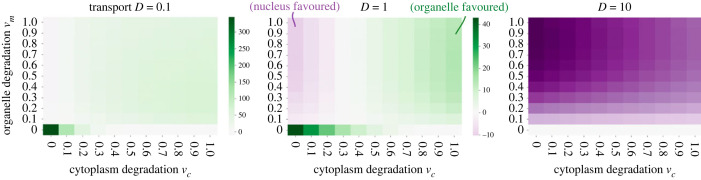
Cost differences with the interplay of degradation and transport in static environments. Colour gives absolute cost difference between nuclear and organelle encoding strategies for a static *E*(*t*) = *a* = 1 environment. The parameter values are synthesis rate *λ* = 1 and wild-type oDNA proportion *p* = 0.75. This proportion *p* influences which compartment is more favourable; results for *p* = 1 and *p* = 0.9 are in electronic supplementary material, figure S3. Randomly varying environments (red and white noise) show very similar profiles of behaviour to this static case (electronic supplementary material, figure S1).

Here, if *C*_0_/*C*_1_ > 1, then encoding the gene in the nucleus is the most favourable strategy, and if *C*_0_/*C*_1_ < 1, then encoding in the organelle is the most favourable strategy. The first condition *C*_0_/*C*_1_ > 1 holds if *D* > *p*(*D* + *ν*_*c*_), which is the case when *D* is high (the import to the organelle is fast), *ν*_*c*_ is low (gene product is not lost in the cytosol so the import to the organelle is more efficient) and *p* is low (the organelle has a significant load of mutant oDNA that prevents the synthesis of gene product). Conversely, the opposite condition *C*_0_/*C*_1_ < 1 holds if *D* < *p*(*D* + *ν*_*c*_), and this is the case when *D* is low (the import to the organelle is slow), *ν*_*c*_ is high (a lot of gene product is degraded and therefore not so much is being imported to the organelle) and *p* is high (there is good proportion of wild-type oDNA to synthesize gene product). These trends are clearly observable in the numerical results in figure 2, illustrating a moderate level of oDNA damage (*p* = 0.75). Without oDNA damage (*p* = 1), there is never a reason to favour the nuclear compartment, and nuclear-encoded costs at best match (but never drop below) organelle-encoded costs. Electronic supplementary material, figure S3 explores higher values of *p*.

Here and throughout, the parameters of the model provide a way of connecting the theory to specific genes. Gene products with high turnover correspond to a high *ν*_*m*_; long-lived products have a low *ν*_*m*_. Gene products prone to mistargeting (which could include hydrophobic products under the ‘mistargeting’ picture [23]) have a low *D*; those challenging to import to the organelle (which could include hydrophobic products under the ‘unfolding’ picture [17]) have a high cytoplasmic loss rate *ν*_*c*_. A value of *p* < 1 corresponds to the expectation that genes encoded in oDNA will accumulate some level of genetic damage over time; lower *p* corresponds to more damage, while higher *p* corresponds to more robust oDNA maintenance [59].

Degradation rates play an important role in the model. The degradation rate in the organelle *ν*_*m*_ limits the ability of the cell to meet environmental demand: the organelle is prevented from having as much gene product as it needs, and the variable *x*_*m*_(*t*) is always damped with respect to an environmental function *E*(*t*). For low *ν*_*m*_ (long-lived gene products), no significant amount of gene product is lost and the total amount in the organelle is more correctly modulated by the signalling function (equation (2.1)). For *ν*_*m*_ = 0, the system can perfectly satisfy environmental demand (electronic supplementary material, text A.4).

### (b) Randomly changing environments

The environmental demands placed on an organism are unlikely to be completely static, and are often modelled as randomly varying [35,60]. To connect with this picture of constant environmental fluctuation, we next explore the effect of randomly varying environmental demands in our model (see Methods). The environments behave either like uncorrelated white noise (equation (2.5)) or like correlated red noise (equation (2.6)). We see in electronic supplementary material, figure S1 that the behaviour for static, uncorrelated and correlated noisy environments show very similar patterns; the same principles for static environmental demands discussed above also hold for noisy ones, except the system is never able to perfectly adapt to the constantly changing environmental demand. This similarity is because the system tends to adapt to an average environmental demand (either a constant for uncorrelated noise or a moving average for red noise) and fluctuations around this adapted average challenge the system in similar ways.

In electronic supplementary material, figure S5, we see that when there is some degradation in the organelle, *ν*_*m*_ = 0.5, and no degradation in the cytosol, *ν*_*c*_ = 0, for uncorrelated white noise the costs for both encoding compartments are almost identical. For correlated red noise, the cost for the nuclear-encoding compartment is lower. These examples agree with the corresponding points in the heatmaps in figure 2 for gene transport rates *D* = 0.1 and *D* = 1.

### (c) Periodically changing environments

**Figure 3.**
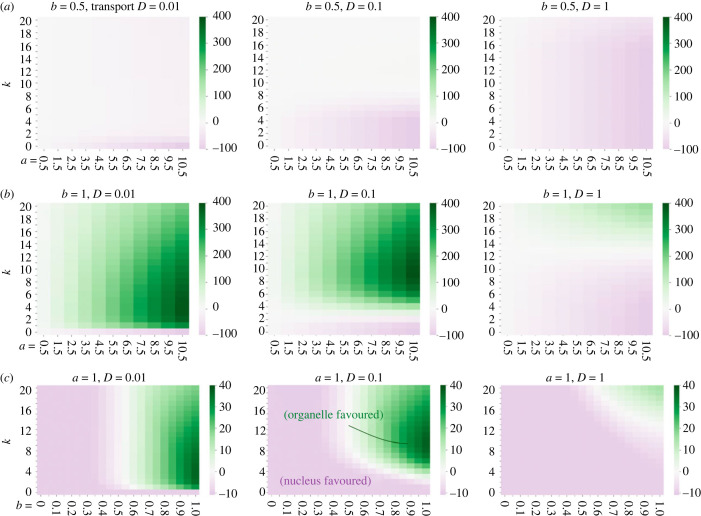
Cost difference with the interplay between environmental oscillation and cell biological processes. Environmental oscillations have frequency *k* per day, time-averaged value *a* (horizontal axis in (*a*) and (*b*)) and relative oscillation amplitude *b* (horizontal axis in (*c*)). Other parameter values are synthesis rate *λ* = 1, degradation in the organelle *ν*_*m*_ = 0.5, no degradation in the cytosol *ν*_*c*_ = 0 and wild-type oDNA proportion *p* = 0.75. Different parameterizations show largely the same trends and are explored in the electronic supplementary material, information: alternative values of *p* (electronic supplementary material, figures S6 and S7); non-zero degradation in the cytosol *ν*_*c*_ = 1 (electronic supplementary material, figures S8 and S10); higher synthesis rate *λ* = 10 (electronic supplementary material, figures S9 and S12); and low synthesis rate *λ* = 0.1 (electronic supplementary material, figure S11).

Next, we consider oscillating environmental demands as defined in equation (2.4). For different frequencies *k* and time-averaged demands *a*, we look at when the oscillation amplitude takes a maximal value (*b* = 1), representing higher-magnitude environment oscillations, and when the amplitude is lower (*b* = 0.5), representing an environment closer to the constant case. Qualitatively, the higher the relative amplitude *b* and the frequency *k*, the more dramatically changing the environment is. We see in figure 3 that encoding the organelle gene in the organelle becomes more favourable the more the environment changes—that is, for high amplitude *b* = 1 and every frequency when the transport is slow (*D* = 0.01, corresponding to hard-to-import gene products), and for high frequencies when the transport is faster (*D* = 0.1 and *D* = 1). This agrees with the hypothesis that organisms tend to retain more genes in their oDNA if their environments dramatically and rapidly change, and suggests that those harder to import (possibly including hydrophobic products [17,23]) will be the most retained.

**Figure 4.**
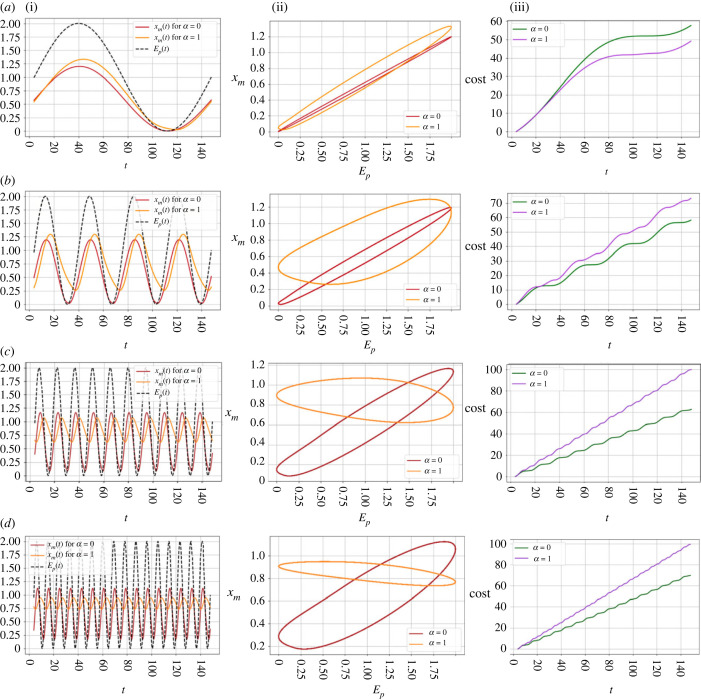
Time behaviour and phase portraits of the system in different examples of periodically changing environments. Different rows give different environmental frequencies *k* progressing vertically up figure 3*c*: (*a*) *k* = 1 (nuclear encoding favoured); (*b*) *k* = 4 (organelle encoding favoured); (*c*) *k* = 10 (organelle encoding most favoured); (*d*) *k* = 16 (organelle encoding less favoured). Left column (i) shows time series of environmental demand *E*(*t*) and *x*_*m*_(*t*) for both encoding compartments. Central column (ii) shows demand versus supply traces; departure from the diagonal corresponds to a supply–demand imbalance. Right column (iii) shows time series of accumulated costs. In each case, the time series are plotted after equilibration (see Methods). The common parameter values in all cases are a synthesis rate *λ* = 1, a degradation rate in the organelle *ν*_*m*_ = 0.5, no degradation in the cytosol *ν*_*c*_ = 0, transport *D* = 0.1, wild-type oDNA proportion *p* = 0.75, time-averaged energetic demand *a* = 1 and relative amplitude *b* = 1.

Strikingly, organelle encoding is most strongly favoured at intermediate frequencies *k* of environmental fluctuation, as seen in figure 3. The specific values of these frequencies depend on the transport rate: for lower transport rates, the value of *k* where organelle is most favoured is lower, and for higher transport rates the value of *k* is higher. Whether this translates to an overall favouring of the organelle compartment also depends on cytoplasmic degradation and oDNA wild-type proportion. This non-monotonic behaviour arises because of different abilities of the system to synchronize with environmental demand, illustrated in figure 4 and demonstrated with calculations in electronic supplementary material, text A.4. For slowly varying environments (figure 4*a*), both encoding strategies can respond reasonably well to changing demand and stay synchronized with the environment, so there is no particular response advantage to oDNA encoding. As the frequency of environmental change *k* increases, synchronicity is lost in both cases, but more so for the nuclear-encoded case (figure 4*b*). At intermediate *k*, the nuclear-encoded case is rather far from synchronization and begins rather to adopt the ‘time-averaged’ picture seen for the noisy environments, with the oscillation amplitude *b* lowering towards a more constant response, while the organelle-encoded case retains some synchronicity (figure 4*c*). At yet higher frequencies (figure 4*d*), the ‘time-averaged’ nuclear-encoded behaviour remains similar but the organelle-encoded case further loses synchronization with the environment, so its relative advantage decreases.

On the other hand, the nuclear-encoding case is most strongly favourable for a less dynamic environment. We see in figure 3 that this compartment begins to dominate for lower amplitude *b* = 0.5, and is completely dominant under these parameterizations for a constant environment (*k* = 0) if the proportion of wild-type oDNA *p* is less than 1. For the case when *p* = 1 (perfect oDNA integrity can be retained), we see in electronic supplementary material, figures S6 and S7 that the nuclear-encoding case is never favoured, by the reasons described above. Following the trends observed in the static environment, decreased organelle damage *p* = 1 (electronic supplementary material, figures S6 and S7), or increased cytoplasmic degradation *ν*_*c*_ = 1 (electronic supplementary material, figures S8 and S10), has the straightforward effect of shifting the trends in figure 3 quite uniformly across parameterizations to favour organelle encoding.

We can again use the parameterizations of the model to consider predicted gene-to-gene behaviour. As an illustration, consider three gene products: one hydrophobic and challenging to import (with *D* = 0.01), one intermediate (with *D* = 0.1) and a less hydrophobic product with less import difficulty (with *D* = 1). Consider also two sets of environmental conditions, *k* = 4 (intermediate frequency) and *k* = 10 (high frequency) with the same *a =* *b =* 1. Under these conditions and the other biologically plausible parameters in figure 3, for intermediate frequency *k* = 4 only the most hydrophobic product (for *D* = 0.01) is favoured in the organelle with the others (for *D* = 0.1 and *D* = 1) favoured in the nucleus. For *k* = 10, only the most hydrophilic (*D* = 1) will remain in the nucleus. Electronic supplementary material, figure S6 also supports this picture for different proportions of wild-type oDNA *p*.

Lastly, from the algebraic solutions of the model in electronic supplementary material, text A.4 and figure 4, we can see explicitly how an oscillatory environment challenges the system, as there is an absence of a perfectly synchronized gene availability solution *x*_*m*_(*t*) for the oscillating environmental demand *E*_*p*_(*t*) (equation (2.4)). This is because for both organelle-encoding and nuclear-encoding cases the only way the system can reach the time-averaged demand *a* in *E*_*p*_(*t*) is if organelle degradation *ν*_*m*_ = 0, but then it cannot enter a decay phase when the environmental wave *E*_*p*_(*t*) goes down. This scenario places the system out-of-phase with respect to the environmental wave, and therefore leads to a high cost. This is seen in figure 4 and electronic supplementary material, figure S2. The expressions derived there show that as environmental frequency *k* increases, synchronization of both the nuclear-encoded and organelle-encoded cases is increasingly challenged. The nuclear-encoded case is consistently more out-of-phase than the organelle-encoded case, but the amplitudes of the system’s responses also change with *k*, leading to the overall monotonic cost behaviour observed here.

### (d) Connection with eukaryotic taxa

In a periodically changing environment (equation (2.4)), the frequency *k* gives the number of environmental oscillations per day: corresponding, for example, to diurnal oscillation if *k* = 1, or to semidiurnal oscillation if *k* = 2. For several plausible parameterizations of genetic properties (rates of expression, import and degradation), these *k* values fall in the region where organelle encoding is most favoured. In particular, organelle encoding is strongly favoured in the face of diurnal or semidiurnal oscillation for genes with low *D*, that is, those that are challenging to import to the organelle (perhaps due to hydrophobicity [17,23]). In such cases, we expect species experiencing strong diurnal or semidiurnal variation in their organelle demands to favour organelle encoding of sets of oDNA genes that appear in nuclear DNA in other species.

To explore the feasibility of this picture, we extracted the distributions of retained gene counts across all eukaryotes with sequenced oDNA (see Methods). Corresponding to our original hypothesis, and the diurnal-semidiurnal predictions of this theory, we asked whether oDNA gene counts in species subjected to such environmental oscillations were higher than those in environments with different (and particularly more limited) dynamics.

**Figure 5.**
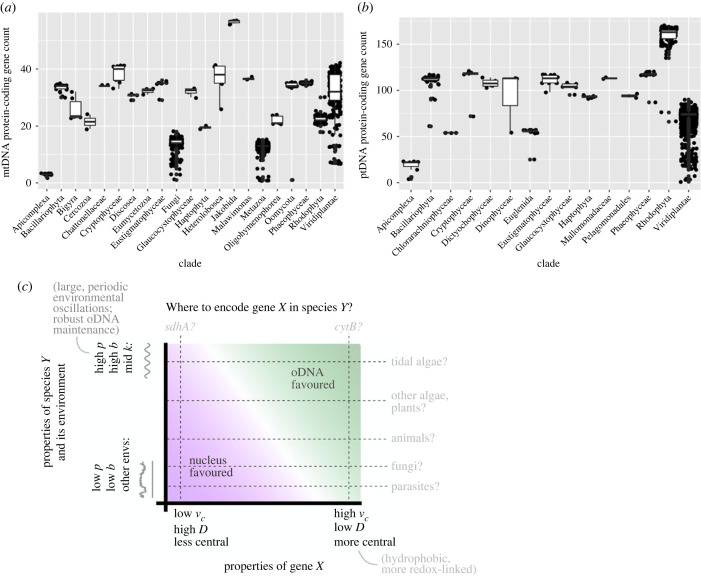
Connecting theory to oDNA profiles of extant eukaryotic taxa. (*a*,*b*) Protein-coding oDNA gene counts in different eukaryotic clades. (*a*) mtDNA, (*b*) ptDNA. The lower points in fungi, metazoa and Viridiplantae (for both organelles) typically correspond to parasitic species, and apicomplexans are all parasites. (*c*) Summary illustration of the key proposed influences suggested by our model. Both cell biological and environmental features influence which compartment is preferred for encoding a given gene: the figure condenses several degrees of freedom into a single illustrative axis in both cases. At the species level, organisms maintaining high genetic integrity (high *p*; assisted, for example, by developmental bottlenecks and recombination [61]) and subject to strong, intermediate frequency environmental oscillations (high *b*, intermediate *k*; for example, diurnal light or tidal oscillations) retain more genes. At the gene level, genes challenging to import to the organelle (high *ν*_*c*_, low *D*; for example, hydrophobic gene products) and playing a more central role in bioenergetic supply [11,62] are the more likely to be retained. Transects through the figure give examples of eukaryotic clades and mtDNA genes (generally oDNA-encoded *cytB* and generally nuclear-encoded *sdhA*) corresponding qualitatively to different cases.

Figure 5*a*,*b* shows the statistics of oDNA gene counts by eukaryotic clade. First, intracellular parasites largely exist inside the highly buffered cells of their host, presented with constant, low demand on their organelles. Correspondingly, they retain very few oDNA genes. This is visible in the mtDNA counts of apicomplexans and subsets of fungi and metazoa. Apicomplexans also possess a plastid-like organelle called the apicoplast, which again contains very few oDNA genes. Free-living fungi often exist on relatively stable nutrient sources like decaying wood or leaf matter (posing few temporal fluctuations in demand), and also retain few mtDNA genes. Plants (within Viridiplantae) are typically subject to diurnal light and temperature changes and, being sessile, cannot move away from other environmental changes, and they retain comparatively many oDNA genes. Sessile, intertidal eukaryotes (including multicellular red and brown algae) face both strong diurnal light fluctuations and semidiurnal tidal variation in oxygen, temperature and salinity, and they retain the highest ptDNA counts [11]. There will certainly be other factors influencing where a given gene is encoded, including the evolutionary history of a species [63], reproductive strategy [25] and many other features. The differing plasticities of organelle gene encoding in different taxa will mean that some species may adopt new encoding strategies on short time scales in response to selective pressures, while some (like metazoans) remain relatively fixed [7]. Another important point is that some genes may have been completely lost from some species rather than transferred from oDNA to the nucleus. In the case of some parasites and other species [24], entire organelle protein complexes have been lost, meaning that the total number of genes to consider in those species is lower than in other species. The majority of species we consider here appear to retain functional copies of the organelle complexes that can contain oDNA-encoded subunits, suggesting that at least a core set of subunits are encoded in one of the two compartments. We do not attempt to control for these complications in a quantitative hypothesis testing framework here, rather we just aim to draw attention to some examples which are compatible with the predictions of our theory.

Our theory suggests that the trade-off between response to environmental demands and maintaining genetic integrity will determine which compartment is favoured for a given gene in a given organism of a specific species. In addition to the different environments described above, different species maintain oDNA integrity to different extents. Plants have lower sequence mutation rates in oDNA than nDNA (although they have pronounced structural diversity in oDNA) [27], due at least in part for their capacity for oDNA recombination [61,64], suggesting that their oDNA integrity levels *p* in our model would be higher and organelle encoding would be further favoured. Metazoa and fungi have high oDNA mutation rates, suggesting a lower *p* and corresponding relative favouring of nuclear encoding.

## 4. Discussion

Our model describes a link between the environmental dynamics that an organism of a given species faces and the extent to which organelle gene retention is favoured (figure 5*c*). Holding other factors equal, it predicts that organisms experiencing high-amplitude and/or intermediate-frequency oscillations in environmental demand will favour oDNA encoding of genes more than organisms in less periodic environments. The scale of the rates and values involved mean that this picture could help explain the high oDNA retention counts of organisms in tidal environments and subject to diurnal light oscillations, and the low retention counts in organisms in buffered, stable, or noisy environments. To our knowledge, this is the first theoretical approach attempting to explain these patterns. Of note is the connection between biophysical features governing the cell biological behaviour of gene products and the ability of the cell to respond to changing environments: a connection between the hydrophobicity [17,23] and CoRR [13,18–21] hypotheses, often viewed as competing [11].

A natural extension to the work presented throughout this paper is to consider oDNA damage inheritance and buildup in an evolutionary time scale, instead of analysing the system for shorter, ecological time scales where the proportion of damaged oDNA 1 − *p* is maintained constant. Another natural target for future work involves the transitions between encoding compartments, as any shift of an oDNA gene to the nucleus will require a transient state in which the gene is encoded in both compartments. Future theoretical work is aimed to address these questions. As with most theories about large-scale evolutionary processes, a direct connection with experimental testing is challenging. It may be possible to experimentally test these ideas further by subjecting otherwise similar organisms that differ in oDNA retention profiles to artificial fluctuating environments and assaying their performance, for example, algae in fluctuating light conditions.

We do not (and cannot) claim that this theory explains the full diversity of oDNA retention profiles across eukaryotes: as with parallel gene-specific questions, it is likely that several factors contribute together. However, although the present-day oDNA gene profile of an organism is shaped by many factors over its entire evolutionary history (including serial endosymbiosis events in the case of plastids [65]), and not just its present-day situation [63], some biological observations do at least qualitatively suggest that the compatibility of gene profiles of modern organisms and their environments does not conflict with our predictions (figure 5). Recent work has shown that the transfer of mtDNA to the nucleus is ongoing in modern humans, estimating that one in every few thousand births experiences a *de novo* transfer of mtDNA material [8]. It is known that transfer of ptDNA to the nucleus occurs frequently even over an individual plant’s lifetime [9]. This ever-present potential for gene transfer suggests that modern organisms’ environments may indeed provide some selective advantage to the oDNA retention profiles they have adopted, and that ongoing selective pressures act to shape oDNA in modern eukaryotes. As oDNA retention patterns for several taxa seem to have been fixed relatively early in evolutionary history [63], our theory can be applied to describe the profiles that were adopted by these ancestral lineages in response to their contemporary environments.

In parallel with this species-specific picture, gene-specific features have been found to rank individual genes from ‘most potential to be retained’ to ‘least potential to be retained’ [11] (figure 5*c*). Those with most retention potential typically encode the most hydrophobic products (corresponding in our model to a high degradation in the cytosol *ν*_*c*_ or slow import of gene product to the organelle *D*, and hence substantial loss in translocation to the organelle), or those most central to the assembly and production of respiratory or photosynthetic complexes (and hence most linked to supplying energetic demands) [11]. This last feature is connected via by the CoRR hypothesis to the retention of organelle genes [21]. Those with least retention potential, by contrast, may experience fewer barriers to translocation and be less essential for fulfilling environmental demands. Taking these pictures together, we can say that species in environments where retention is disfavoured will only retain those gene with highest retention potential, whereas species in environments where retention is more favoured will retain more of the lower-potential genes (figure 5*c*). Our model thus connects cell biology, environmental demands and genetic location to propose how specific combinations of gene and species properties shape the patterns of oDNA retention across species.

## Data Availability

All data and code is publicly available at https://github.com/StochasticBiology/Environmental-oDNA-retention.git. The data are provided in the electronic supplementary material [66].

## References

[1] Adams KL, Palmer JD. 2003 Evolution of mitochondrial gene content: gene loss and transfer to the nucleus. Mol. Phylogenet. Evol. **29**, 380-395. (10.1016/S1055-7903(03)00194-5) 14615181

[2] Martin WF, Garg S, Zimorski V. 2015 Endosymbiotic theories for eukaryote origin. Phil. Trans. R. Soc. B **370**, 20140330. (10.1098/rstb.2014.0330) 26323761 PMC4571569

[3] Roger AJ, Muñoz-Gómez SA, Kamikawa R. 2017 The origin and diversification of mitochondria. Curr. Biol. **27**, R1177-R1192. (10.1016/j.cub.2017.09.015) 29112874

[4] Sagan L. 1967 On the origin of mitosing cells. J. Theor. Biol. **14**, 225–IN6. (10.1016/0022-5193(67)90079-3) 11541392

[5] Sloan DB, Warren JM, Williams AM, Wu Z, Abdel-Ghany SE, Chicco AJ, Havird JC. 2018 Cytonuclear integration and co-evolution. Nat. Rev. Genet. **19**, 635-648. (10.1038/s41576-018-0035-9) 30018367 PMC6469396

[6] Zaremba-Niedzwiedzka K et al. 2017 Asgard archaea illuminate the origin of eukaryotic cellular complexity. Nature **541**, 353-358. (10.1038/nature21031) 28077874

[7] Timmis JN, Ayliffe MA, Huang CY, Martin W. 2004 Endosymbiotic gene transfer: organelle genomes forge eukaryotic chromosomes. Nat. Rev. Genet. **5**, 123-135. (10.1038/nrg1271) 14735123

[8] Wei W, Schon KR, Elgar G, Orioli A, Tanguy M, Giess A, Tischkowitz M, Caulfield MJ, Chinnery PF. 2022 Nuclear-embedded mitochondrial DNA sequences in 66 083 human genomes. Nature **611**, 105-114. (10.1038/s41586-022-05288-7) 36198798 PMC9630118

[9] Stegemann S, Hartmann S, Ruf S, Bock R. 2003 High-frequency gene transfer from the chloroplast genome to the nucleus. Proc. Natl Acad. Sci. USA **100**, 8828-8833. (10.1073/pnas.1430924100) 12817081 PMC166398

[10] Daley DO, Whelan J. 2005 Why genes persist in organelle genomes. Genome Biol. **6**, 110. (10.1186/gb-2005-6-5-110) 15892877 PMC1175947

[11] Giannakis K, Arrowsmith SJ, Richards L, Gasparini S, Chustecki JM, Røyrvik EC, Johnston IG. 2022 Evolutionary inference across eukaryotes identifies universal features shaping organelle gene retention. Cell Syst. **3**, 874-884. (10.1016/j.cels.2022.08.007) 36115336

[12] Johnston IG, Williams BP. 2016 Evolutionary inference across eukaryotes identifies specific pressures favoring mitochondrial gene retention. Cell Syst. **2**, 101-111. (10.1016/j.cels.2016.01.013) 27135164

[13] Allen JF, Raven JA. 1996 Free-radical-induced mutation vs redox regulation: costs and benefits of genes in organelles. J. Mol. Evol. **42**, 482-492. (10.1007/BF02352278) 8662000

[14] Kennedy SR, Salk JJ, Schmitt MW, Loeb LA. 2013 Ultra-sensitive sequencing reveals an age-related increase in somatic mitochondrial mutations that are inconsistent with oxidative damage. PLoS Genet. **9**, 1-10. (10.1371/journal.pgen.1003794) PMC3784509 24086148

[15] Itsara LS, Kennedy SR, Fox EJ, Yu S, Hewitt JJ, Sanchez-Contreras M, Cardozo-Pelaez F, Pallanck LJ. 2014 Oxidative stress is not a major contributor to somatic mitochondrial DNA mutations. PLoS Genet. **10**, 1-13. (10.1371/journal.pgen.1003974) PMC3916223 24516391

[16] Blanchard JL, Lynch M. 2000 Organellar genes: why do they end up in the nucleus? Trends Genet. **16**, 315-320. (10.1016/S0168-9525(00)02053-9) 10858662

[17] von Heijne G. 1986 Why mitochondria need a genome. FEBS Lett. **198**, 1-4. (10.1016/0014-5793(86)81172-3) 3514271

[18] Allen J. 2003 The function of genomes in bioenergetic organelles. Phil. Trans. R. Soc. Lond. B **358**, 19-37. (10.1098/rstb.2002.1191) 12594916 PMC1693096

[19] Allen J. 2003 Why chloroplasts and mitochondria contain genomes. Comp. Funct. Genom. **4**, 31-36. (10.1002/cfg.245) PMC2447392 18629105

[20] Allen J. 1993 Control of gene expression by redox potential and the requirement for chloroplast and mitochondrial genomes. J. Theor. Biol. **165**, 609-631. (10.1006/jtbi.1993.1210) 8114509

[21] Allen J. 2015 Why chloroplasts and mitochondria retain their own genomes and genetic systems: colocation for redox regulation of gene expression. Proc. Natl Acad. Sci. USA **112**, 10 231-10 238. (10.1073/pnas.1500012112) 26286985 PMC4547249

[22] Popot J-L, de Vitry C. 1990 On the microassembly of integral membrane proteins. Annu. Rev. Biophys. Biophys. Chem. **19**, 369-403. (10.1146/annurev.bb.19.060190.002101) 2194481

[23] Björkholm P, Harish A, Hagström E, Ernst AM, Andersson SGE. 2015 Mitochondrial genomes are retained by selective constraints on protein targeting. Proc. Natl Acad. Sci. USA **112**, 10 154-10 161. (10.1073/pnas.1421372112) 26195779 PMC4547212

[24] Hjort K, Goldberg AV, Tsaousis AD, Hirt RP, Embley TM. 2010 Diversity and reductive evolution of mitochondria among microbial eukaryotes. Phil. Trans. R. Soc. B **365**, 713-727. (10.1098/rstb.2009.0224) 20124340 PMC2817227

[25] Brandvain Y, Barker MS, Wade MJ. 2007 Gene co-inheritance and gene transfer. Science **315**, 1685-1685. (10.1126/science.1134789) 17379800

[26] Brandvain Y, Wade MJ. 2009 The functional transfer of genes from the mitochondria to the nucleus: the effects of selection, mutation, population size and rate of self-fertilization. Genetics **182**, 1129-1139. (10.1534/genetics.108.100024) 19448273 PMC2728854

[27] Johnston IG. 2019 Tension and resolution: dynamic, evolving populations of organelle genomes within plant cells. Mol. Plant **12**, 764-783. (10.1016/j.molp.2018.11.002) 30445187

[28] Hadjivasiliou Z, Lane N, Seymour RM, Pomiankowski A. 2013 Dynamics of mitochondrial inheritance in the evolution of binary mating types and two sexes. Proc. R. Soc. B **280**, 20131920. (10.1098/rspb.2013.1920) PMC3768323 23986113

[29] Hadjivasiliou Z, Pomiankowski A, Seymour RM, Lane N. 2012 Selection for mitonuclear co-adaptation could favour the evolution of two sexes. Proc. R. Soc. B **279**, 1865-1872. (10.1098/rspb.2011.1871) PMC3297446 22158961

[30] Radzvilavicius A, Layh S, Hall MD, Dowling DK, Johnston IG. 2021 Sexually antagonistic evolution of mitochondrial and nuclear linkage. J. Evol. Biol. **34**, 757-766. (10.1111/jeb.13776) 33644926

[31] Radzvilavicius AL, Hadjivasiliou Z, Pomiankowski A, Lane N. 2016 Selection for mitochondrial quality drives evolution of the germline. PLoS Biol. **14**, e2000410. (10.1371/journal.pbio.2000410) 27997535 PMC5172535

[32] Radzvilavicius AL, Lane N, Pomiankowski A. 2017 Sexual conflict explains the extraordinary diversity of mechanisms regulating mitochondrial inheritance. BMC Biol. **15**, 1-12. (10.1186/s12915-017-0437-8) 29073898 PMC5658935

[33] Kelly S. 2021 The economics of organellar gene loss and endosymbiotic gene transfer. Genome Biol. **22**, 345. (10.1186/s13059-021-02567-w) 34930424 PMC8686548

[34] Hoitzing H, Gammage PA, Haute LV, Minczuk M, Johnston IG, Jones NS. 2019 Energetic costs of cellular and therapeutic control of stochastic mitochondrial dna populations. PLoS Comput. Biol. **15**, 1-27. (10.1371/journal.pcbi.1007023) PMC6615642 31242175

[35] Vasseur D, Yodzis P. 2004 The color of environmental noise. Ecology **85**, 1146-1152. (10.1890/02-3122)

[36] Johnston I, Rickett B, Jones N. 2014 Explicit tracking of uncertainty increases the power of quantitative rule-of-thumb reasoning in cell biology. Biophys. J. **107**, 2612-2617. (10.1016/j.bpj.2014.08.040) 25468340 PMC4255194

[37] Pelechano V, Chávez S, Pérez-Ortín JE. 2010 A complete set of nascent transcription rates for yeast genes. PLoS ONE **5**, e15442. (10.1371/journal.pone.0015442) 21103382 PMC2982843

[38] Siwiak M, Zielenkiewicz P. 2010 A comprehensive, quantitative, and genome-wide model of translation. PLoS Comput. Biol. **6**, e1000865. (10.1371/journal.pcbi.1000865) 20686685 PMC2912337

[39] Belle A, Tanay A, Bitincka L, Shamir R, O’Shea EK. 2006 Quantification of protein half-lives in the budding yeast proteome. Proc. Natl Acad. Sci. USA **103**, 13 004-13 009. (10.1073/pnas.0605420103) PMC1550773 16916930

[40] Christiano R, Nagaraj N, Fröhlich F, Walther TC. 2014 Global proteome turnover analyses of the yeasts *S. cerevisiae* and *S. pombe*. Cell Rep. **9**, 1959-1965. (10.1016/j.celrep.2014.10.065) 25466257 PMC4526151

[41] Schäfer JA, Bozkurt S, Michaelis JB, Klann K, Münch C. 2022 Global mitochondrial protein import proteomics reveal distinct regulation by translation and translocation machinery. Mol. Cell **82**, 435-446. 34847359 10.1016/j.molcel.2021.11.004 PMC8791276

[42] Li L, Nelson C, Fenske R, Trösch J, Pružinská A, Millar AH, Huang S. 2017 Changes in specific protein degradation rates in Arabidopsis thaliana reveal multiple roles of Lon1 in mitochondrial protein homeostasis. Plant J. **89**, 458-471. (10.1111/tpj.13392) 27726214

[43] Guo Y, Li C-I, Sheng Q, Winther JF, Cai Q, Boice JD, Shyr Y. 2013 Very low-level heteroplasmy mtdna variations are inherited in humans. J. Genet. Genomics **40**, 607-615. (10.1016/j.jgg.2013.10.003) 24377867 PMC4149221

[44] Johnston IG. 2019 Varied mechanisms and models for the varying mitochondrial bottleneck. Front. Cell Dev. Biol. **7**, 294. (10.3389/fcell.2019.00294) 31824946 PMC6879659

[45] Hindmarsh A. 1983 Odepack, a systematized collection of ODE solvers. In Scientific computing (eds RS Stepleman et al.), pp. 55-64. Amsterdam, The Netherlands: North-Holland.

[46] Radhakrishnan K, Hindmarsh A. 1993 Description and use of lsode, the Livermore solver for ordinary differential equations, technical report UCRL-ID-113855. Livermore, CA: Lawrence Livermore National Laboratory.

[47] Waskom ML. 2021 seaborn: statistical data visualization. J. Open Sourc. Softw. **6**, 3021. (10.21105/joss.03021)

[48] Hunter JD. 2007 Matplotlib: a 2D graphics environment. Comput. Sci. Eng. **9**, 90-95. (10.1109/MCSE.2007.55)

[49] Harris CR et al. 2020 Array programming with NumPy. Nature **585**, 357-362. (10.1038/s41586-020-2649-2) 32939066 PMC7759461

[50] Johansson F et al. 2013 *mpmath: a Python library for arbitrary-precision floating-point arithmetic (version 0.18)*. See http://mpmath.org/.

[51] O’Leary NA et al. 2015 Reference sequence (RefSeq) database at NCBI: current status, taxonomic expansion, and functional annotation. Nucleic Acids Res. **44**, D733-D745. 26553804 10.1093/nar/gkv1189 PMC4702849

[52] Cock PJA et al. 2009 Biopython: freely available Python tools for computational molecular biology and bioinformatics. Bioinformatics **25**, 1422-1423. (10.1093/bioinformatics/btp163) 19304878 PMC2682512

[53] Federhen S. 2012 The NCBI taxonomy database. Nucleic Acids Res. **40**(Database issue), D136-D143. (10.1093/nar/gkr1178) 22139910 PMC3245000

[54] R Core Team. 2020 R: a language and environment for statistical computing. Vienna, Austria: R Foundation for Statistical Computing.

[55] Wickham H 2016 ggplot2: elegant graphics for data analysis. New York, NY: Springer.

[56] Auguie B. 2017 *gridExtra: Miscellaneous Functions for ‘Grid’ Graphics*. R package version 2.3.

[57] Revell LJ. 2012 phytools: an R package for phylogenetic comparative biology (and other things). Methods Ecol. Evol. **3**, 217-223. (10.1111/j.2041-210X.2011.00169.x)

[58] Milo R, Phillips R. 2015 Cell biology by the numbers. New York, NY: Garland Science.

[59] Johnston IG, Burgstaller JP. 2019 Evolving mtdna populations within cells. Biochem. Soc. Trans. **47**, 1367-1382. (10.1042/BST20190238) 31484687 PMC6824680

[60] Bell G. 2010 Fluctuating selection: the perpetual renewal of adaptation in variable environments. Phil. Trans. R. Soc. B **365**, 87-97. (10.1098/rstb.2009.0150) 20008388 PMC2842698

[61] Edwards DM, Røyrvik EC, Chustecki JM, Giannakis K, Glastad RC, Radzvilavicius AL, Johnston IG. 2021 Avoiding organelle mutational meltdown across eukaryotes with or without a germline bottleneck. PLoS Biol. **19**, e3001153. (10.1371/journal.pbio.3001153) 33891583 PMC8064548

[62] Allen JF, Martin WF. 2016 Why have organelles retained genomes? Cell Syst. **2**, 70-72. (10.1016/j.cels.2016.02.007) 27135161

[63] Janouškovec J, Tikhonenkov DV, Burki F, Howe AT, Rohwer FL, Mylnikov AP, Keeling PJ. 2017 A new lineage of eukaryotes illuminates early mitochondrial genome reduction. Curr. Biol. **27**, 3717-3724. (10.1016/j.cub.2017.10.051) 29174886

[64] Chen XJ. 2013 Mechanism of homologous recombination and implications for aging-related deletions in mitochondrial dna. Microbiol. Mol. Biol. Rev. **77**, 476-496. (10.1128/MMBR.00007-13) 24006472 PMC3811608

[65] Keeling PJ. 2010 The endosymbiotic origin, diversification and fate of plastids. Phil. Trans. R. Soc. B **365**, 729-748. (10.1098/rstb.2009.0103) 20124341 PMC2817223

[66] Pascual BG, Nordbotten JM, Johnston IG. 2023 Cellular and environmental dynamics influence species-specific extents of organelle gene retention. Figshare. (10.6084/m9.figshare.c.6440229) PMC9993063 36883279

